# Viral lower respiratory tract infections—strict admission guidelines for young children can safely reduce admissions

**DOI:** 10.1007/s00431-021-04057-4

**Published:** 2021-04-08

**Authors:** Lise Beier Havdal, Britt Nakstad, Hans Olav Fjærli, Christian Ness, Christopher Inchley

**Affiliations:** 1 Department of Pediatric and Adolescent Medicine, Akershus University Hospital, Sykehusveien 25, 1478, Nordbyhagen, Norway; 2 Division of Paediatric and Adolescent Medicine, Institute of Clinical Medicine, University of Oslo, Oslo, Norway; 3 Institute of Clinical Medicine, University of Oslo, Oslo, Norway

**Keywords:** Bronchiolitis, Child, Preschool, Hospitalization, Clinical medicine, Emergency service

## Abstract

Viral lower respiratory tract infection (VLRTI) is the most common cause of hospital admission among small children in high-income countries. Guidelines to identify children in need of admission are lacking in the literature. In December 2012, our hospital introduced strict guidelines for admission. This study aims to retrospectively evaluate the safety and efficacy of the guidelines. We performed a single-center retrospective administrative database search and medical record review. ICD-10 codes identified children < 24 months assessed at the emergency department for VLRTI for a 10-year period. To identify adverse events related to admission guidelines implementation, we reviewed patient records for all those discharged on primary contact followed by readmission within 14 days. During the study period, 3227 children younger than 24 months old were assessed in the ED for VLRTI. The proportion of severe adverse events among children who were discharged on their initial emergency department contact was low both before (0.3%) and after the intervention (0.5%) (*p*=1.0). Admission rates before vs. after the intervention were for previously healthy children > 90 days 65.3% vs. 53.3% (*p*<0.001); for healthy children ≤ 90 days 85% vs. 68% (*p*<0.001); and for high-risk comorbidities 74% vs. 71% (*p*=0.5).

*Conclusion*: After implementation of admission guidelines for VLRTI, there were few adverse events and a significant reduction in admissions to the hospital from the emergency department. Our admission guidelines may be a safe and helpful tool in the assessment of children with VLRTI.

**Table:** 

**What is Known:** *• Viral lower respiratory tract infection, including bronchiolitis, is the most common cause of hospitalization for young children in the developed world. Treatment is mainly supportive, and hospitalization should be limited to the cases in need of therapeutic intervention.* *• Many countries have guidelines for the management of the disease, but the decision on whom to admit for inpatient treatment is often subjective and may vary even between physicians in the same hospital.*	
**What is New:** *• Implementation of admission criteria for viral lower respiratory tract infection may reduce the rate of hospital admissions without increasing adverse events.*

**Supplementary Information:**

The online version contains supplementary material available at 10.1007/s00431-021-04057-4.

## Introduction

Viral lower respiratory tract infection (VLRTI) including bronchiolitis and viral pneumonia is the leading cause of hospitalization in children under 24 months of age in high-income countries [1–6]. It is a clinical diagnosis, with the main clinical features being coryza, poor feeding, difficulty breathing, cough, wheeze, and crepitations on auscultation [2, 3, 7]. Management of VLRTI is primarily supportive, with no effective therapies available [1, 2, 8, 9]. There is iatrogenic risk connected to hospitalization, due to medical management errors [10] and nosocomial infections [11, 12]. In addition, one study indicated that excessive handling of children hospitalized for VLTRI can increase both the length of stay and the need for supportive care [13]. Treatment should therefore promote minimal handling, and hospitalization should be limited to cases in need of therapeutic intervention.

Despite the prevalence of the disease, the emergency department (ED) decision to hospitalize is often subjective and may therefore vary between physicians in the same hospital [14]. While many countries have guidelines for the management of VLRTI [1, 15–17], there are few guidelines for when hospital admission is necessary [18].

Several studies have developed prognostic criteria for patients with bronchiolitis, indicating which patients should be admitted [3, 15, 19–25] and leading to clinical risk score models that have been tested retrospectively for accuracy [26–28]. However, the models usually assume that the ED decision to admit the patient was always appropriate, using this as the outcome measure. Inter-physician variability may thus limit the usefulness of such models. Lou et al. developed and studied an operational definition of appropriate hospital admission for bronchiolitis, which may be more objective: ≥ 6-h exposure to major medical interventions defined a necessary hospital admission; return to the ED within 12 h followed by hospitalization defined an unsafe ED discharge [29].

Studying patient data in our hospital, we found that many infants hospitalized with bronchiolitis did not receive any supportive therapy but were admitted for observation only. We saw a potential to improve our practice by the development of guidelines for patient admission.

The primary aim of this study was to evaluate the safety of the guidelines in previously healthy children aged > 90 days to < 24 months presenting with VLRTI. Secondary aims were to (i) assess safety in children aged ≤ 90 days, or with medical conditions predisposing to more severe VLRTI, and (ii) determine the guidelines’ efficacy to change hospital admission rates, return rates, or use of major medical interventions.

## Materials and methods

This study is a retrospective analysis of pediatric admissions and re-admissions for VLRTI before and after the implementation of strict admission guidelines.

Admission criteria were based on both literature review and clinical information from a prospective study conducted on children assessed for viral airway infection in our ED [3, 30, 31]. Before implementation, the guidelines were discussed with the staff in both the ED and the inpatient department (IPD), including attending physicians and nurses.

In December 2012, these guidelines were implemented in the pediatric ED at Akershus University Hospital, with strict and precise criteria for admission to the IPD.

**Fig. 1:**
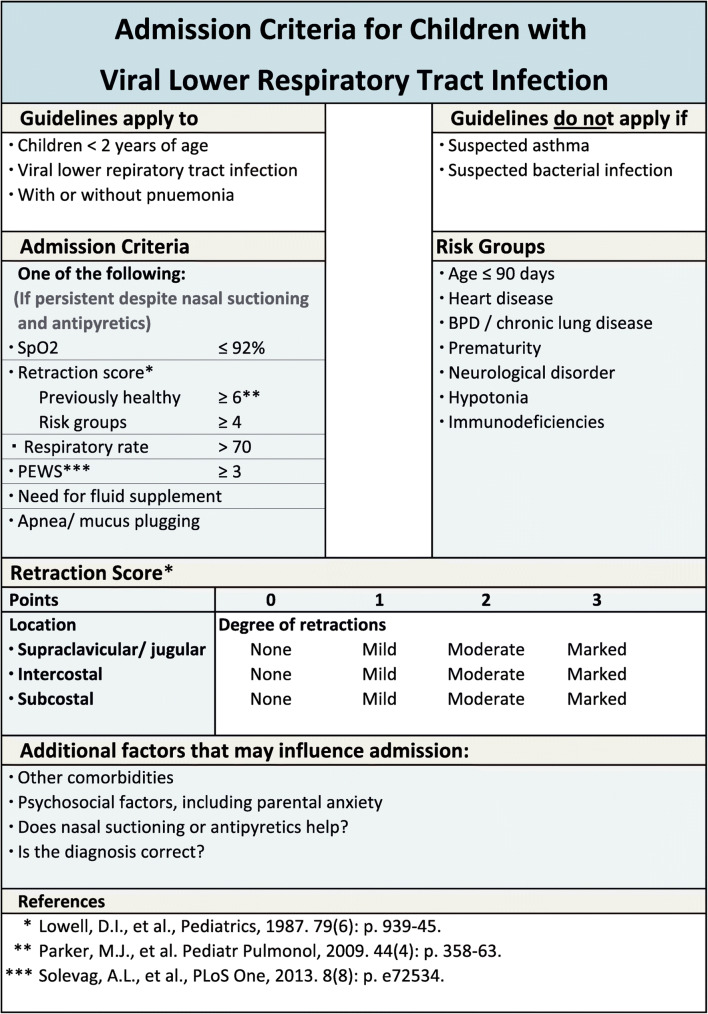
Admission criteria for children with viral lower respiratory tract infection. PEWS, Pediatric Early Warning Score

The admission guidelines with modifying risk factors are presented in Fig. 1 and apply to children less than 2 years of age with VLRTI with or without pneumonia, including children with a first episode of wheeze. The criteria do not apply to children with suspicion of bacterial infection or asthma.

To avoid short-term improvements in clinical parameters that might affect the decision to admit or send home, ED staff were discouraged from giving nebulized saline or bronchodilators to children awaiting a decision on admission.

As part of the intervention, parents of any child not admitted to the in-patient department were educated in management of their sick child before leaving the hospital. All parents were provided with a manual nasal aspirator (vacuum provided by oral suctioning) and trained in nasal suctioning of the child by the following procedure: (i) saline drops in each nostril 1 to 2 min before suctioning; (ii) gentle suctioning from the anterior nares; (iii) deep nasal suctioning discouraged; and (iv) procedure repeated as needed, ideally before feeding, maximum 8 times daily. Parents were encouraged to apply oxymetazoline drops up to three times daily. Parents were not routinely provided with nebulizers or similar devices for inhalation of saline or bronchodilators.

Admission criteria were implemented from December 1, 2012–February 1, 2013. This period is defined as the intervention, and patients admitted during this period were excluded from the study, to acknowledge that not all medical staff had become familiar with the new procedures. January 1, 2009–November 30, 2012, was defined as pre-intervention. February 2, 2013–December 31, 2019, was defined as post-intervention. During the intervention, authors CI and HOF conducted a 2-month period of intensive education of junior and senior doctors and nurses on the ED and demanded details of how guidelines were applied for each patient admitted from the ED. Guidelines were published on posters and in pocket format and made easily available to all staff and presented regularly during subsequent years as a part of the departmental teaching program. Author CI monitored patients who returned to the ED during implementation for serious adverse events.

Concurrently with admission guidelines implementation, we introduced guidelines for treatment and discharge of children with VLRTI. Treatment guidelines included the following: (i) promotion of minimal handling; (ii) restriction of supplementary oxygen to children with SpO2 < 92% for more than 2 h; (iii) children with SpO2 < 88% or cyanosis were given supplemental oxygen immediately; (iv) bronchodilators were discouraged; (v) nebulized saline was given on demand, rather than by fixed-schedule [13]; and (vi) non-invasive respiratory support was reserved for children with severe respiratory distress. Discharge criteria were as follows: (i) no need for supplemental oxygen the last 6 h; (ii) not clinically dehydrated; (iii) adequate fluid intake and urine production; (iv) parents have been trained in nasal suctioning as described above; and (v) no expectation of deterioration in condition, in particular if still early during the illness. Signs of respiratory distress alone were not considered a reason for continued admission if all other parameters were adequate.

### Hospital setting

Akershus University Hospital serves a child population of approximately 125,000 children, of whom around 12,000 are younger than 2 years of age. The pediatric ED is a secondary health-care center, and any visit requires referral from a general practitioner or primary health-care emergency ward. The Norwegian public health-care system is organized in health districts, with clearly defined hospital catchment areas. There are no private hospitals providing emergency care for children. This ensures that unless patients travel to another part of the country during their illness, (i) patients living in the hospital’s catchment area are not referred to another hospital for emergency care and (ii) any child would return to the same ED if in need of a second visit. In addition, comorbidities associated with severe VLRTI would in almost all cases be known to the hospital prior to admission with VLRTI.

### Patient identification and characteristics

Data collection was conducted in September 2019. The data capture group at Akershus University Hospital accessed the hospital’s patient database, identifying children aged ≤ 24 months with an episode of care for VLRTI, defined by ICD-10 discharge codes (Online Resource 1), between January 2008 and August 2019.

For each patient, we collected background data (sex, age, comorbidities) and contact-specific data (length of stay, hospital readmission within 14 days of the primary contact). ICD-10 codes from any previous hospital contacts identified comorbidities (Online Resource 2).

To assess guidelines safety, (i) author LH reviewed electronic health records of all children admitted to the IPD within 14 days of discharge from the ED or IPD, determining if the readmission was due to the same episode of VLRTI. Adverse events and serious adverse events at the time of return were identified. Adverse events were defined as a patient returning in need of oxygen supplement or nasogastric fluid supplements administered within short time after arrival. Serious adverse events were defined as any child immediately given either intravenous fluids or respiratory support (high-flow nasal cannula (HFNC), continuous positive airway pressure (CPAP), or bi-phasic positive airway pressure (BiPAP)) on readmission. (ii) We calculated readmission rates within 12 h, considering this as inappropriate discharge [27]. (iii) We compared overall return rates before and after the intervention.

To evaluate criteria efficacy, (i) we compared the overall rate of admission to the IPD on initial contact and for patients who were reassessed on the ED for the same episode of VLRTI. (ii) Author LH reviewed health records for major medical interventions (MMIs) in a subset of 160 randomly selected patients admitted to the IPD: 80 before and 80 after the intervention. MMI was defined as supplementary oxygen, rehydration (intravenous or by tube feeding), or any form of invasive or non-invasive respiratory support.

### Statistical analysis

Statistical analysis was performed using IBM SPSS version 25 and Stata version 16. Significance levels were 2-sided and set at *p*<0.05. Proportions were analyzed with Fischer’s exact test. Continuous data was analyzed using independent samples median test. For patients discharged from the ED on their initial visit, recontacts were plotted as Kaplan-Meier estimates and analyzed using log-rank test. Patients returning to the ED for reasons other than VLRTI were censored. All patients were censored at 360 h after the initial contact. Population-adjusted means of ED visits and IPD admissions were calculated by season (July 1, 2008, to June 30, 2009, etc.). As the number of observations was small, Mann-Whitney U test was used for comparison. Interrupted time series analysis was performed on admission rates, using segmented regression analysis. The first half of 2008 was an extreme outlier regarding the admission rate for VLRTI. The hospital moved to another location September 2008, possibly altering the hospital database. We were not able to identify or adjust for this; therefore admissions during the first half of 2008 were removed from population-based estimates, and admissions from the whole of 2008 removed from detailed analysis.

### Ethics

The purpose of this study was quality improvement. The hospital’s data protection officer approved the study without need for informed consent or approval from the regional ethics committee.

## Results

From January 1, 2009, to August 21, 2019, 3416 children under 2 years of age received a discharge diagnosis of VLRTI at Akershus University Hospital. The period from December 1, 2012, to February 1, 2013, was considered the intervention period during which guidelines were implemented. During the implementation period, 189 patients were treated and therefore excluded, leaving a total of 3227 children for analysis, 1136 before the intervention and 2091 after. Of these, the number of previously healthy children before the intervention was 753 (66%) and 1236 (60%) after the intervention. Baseline characteristics of the study population are listed in Table 1.

**Table 1:** Baseline characteristics of the study population

	Before intervention *N* (% of total)		After intervention *N* (% of total)		*p* value^1^
All children					
Total	1136	(35%)	2091	(65%)	
Age in days, median (interquartile range)	206	(93–398)	196	(79–428)	0.29^2^
Male sex (*n*=1907)	669	(59%)	1238	(59%)	0.9
Previously healthy, age ≤ 90 d	256	(23%)	559	(27%)	0.01
Prematurity^3^	82	(7.2%)	179	(8.6%)	0.2
Congenital heart disease^3^	45	(4.0%)	81	(3.9%)	0.9
Neuromuscular impairment^3^	10	(0.9%)	34	(1.6%)	0.11
Pulmonary disease^3^	19	(1.7%)	33	(1.6%)	0.9
Immunosuppression^3^	3	(0.3%)	9	(0.4%)	0.6
Previously healthy, > 90 d	753	(66%)	1236	(60%)	0.001
Age in days, median (interquartile range)	307	(174–467)	329	(182–506)	0.19^2^
Male sex (*n*=1221)	452	(60%)	769	(61%)	0.7
Patients selected for medical record review	80	-	80	-	-
Male sex	44	(55%)	44	(55%)	1.00
Age in days, median (interquartile range)	204	(92–430)	332	(61–513)	0.21^2^
Previously healthy, age ≤ 90 d	17	(21%)	28	(35%)	0.08
Any comorbidity^4^	11	(13.8%)	9	(11.3%)	0.81

^1^Fisher’s exact test, unless otherwise stated

^2^Independent samples median test

^3^Risk factors for severe disease based on ICD-10 codes registered for the patient before the episode of VLRTI, as listed in Appendix 1

^4^Comorbidities: prematurity, congenital heart disease, neuromuscular impairment, pulmonary disease, or immunosuppression

### Adverse events

Eighteen children discharged from the ED after initial assessment experienced an adverse event, with similar proportions before and after the intervention, as described in Table 2. Among previously healthy children older than 90 days, non-severe adverse events occurred in 2 (0.8%) children before and 2 (0.3%) after the intervention (*p*= 0.6%), and serious adverse events in 0 (0.0%) cases before and 2 (0.3%) after the intervention (*p*=1.0). There were similar results for children ≤ 90 days and those with comorbidities.

**Table 2:** Emergency department contacts, inpatient department admissions, and adverse events before and after implementation of admission guidelines for children under 2 years of age with viral lower respiratory tract infection

	Before guidelines		After guidelines		*p* value^a^
Population-adjusted contacts for VLRTI^b^	30 per 1000		26 per 1000		0.5^c^
Population-adjusted admissions for VLRTI^b^	23 per 1000		16 per 1000		0.019^c^
Inpatient department admissions on initial emergency department contact					
All children	804/1136	(70.8%)	1244/2091	(59.5%)	<0.001
Previously healthy, age > 90 d	492/753	(65.3%)	673/1263	(53.3%)	<0.001
Previously healthy, age ≤ 90 d	218/256	(85.2%)	380/559	(68.0%)	<0.001
Comorbidity	94/127	(74.0%)	191/269	(71.0%)	0.5
Recontact within 14 days^d^, children not admitted on first ED contact					
All children	63/332	(19.0%)	134/847	(15.8%)	0.19
Previously healthy, age > 90 d	46/261	(17.6%)	73/590	(12.4%)	0.053
Previously healthy, age ≤ 90 d	7/38	(18.4%)	54/179	(30.2%)	0.17
Comorbidity	10/33	(30.3%)	7/78	(9.0%)	0.008
Inpatient department admission within 14 days^d^, children not admitted on first ED contact					
All children	36/332	(10.8%)	57/847	(6.7%)	0.022
Previously healthy, age > 90 d	24/261	(9.2%)	26/590	(4.4%)	0.011
Previously healthy, age ≤ 90 d	5/38	(13.2%)	27/179	(15.1%)	1.0
Comorbidity	7/33	(21.2%)	4/78	(5.1%)	0.015
Recontact within 12 h, children not admitted on first ED contact					
All children	9/332	(2.7%)	19/847	(2.2%)	0.7
Inpatient department admission within 12 h, children not admitted on first ED contact					
All children	4/332	(1.2%)	10/847	(1.2%)	1.0
Non-serious adverse event on recontact^e^					
All children	4/332	(1.2%)	7/847	(0.8%)	0.5
Previously healthy, age > 90 d	2/261	(0.8%)	2/590	(0.3%)	0.6
Previously healthy, age ≤ 90 d	1/38	(2.6%)	5/179	(2.8%)	1.0
Comorbidity	1/33	(3.0%)	0/78	(0.0%)	0.3
Serious adverse event on recontact^f^					
All children	1/332	(0.3%)	4/847	(0.5%)	1.0
Previously healthy, age > 90 d	0/261	(0.0%)	2/590	(0.3%)	1.0
Previously healthy, age ≤ 90 d	1/38	(2.6%)	2/179	(1.1%)	0.4
Comorbidity	0/33	(0.0%)	0/78	(0.0%)	
Major medical intervention, all children^g^	36/80	(45%)	57/80	(71%)	0.001
Major medical intervention, previously healthy, age > 90 d	22/52	(42%)	33/43	(76%)	0.001

^a^Fisher’s exact test, unless otherwise indicated

^b^Mean of population-adjusted rates calculated for each season, based on the average size of the population younger than 2 years of age in the catchment area of the hospital during the same season

^c^Mann-Whitney U test

^d^Children with recontact for causes other than respiratory infection was not counted as recontacts

^e^Non-serious adverse event is defined as immediate need for nasogastric feeding or oxygen supplementation upon recontact

^f^Serious adverse event is defined as immediate need of intravenous rehydration, HHHFNC, CPAP, BiPAP, or intubation on readmission

^g^Based on medical health record review of 160 randomly chosen children. Major medical interventions: nasogastric or intravenous fluids, oxygen supplement or HHHFNC, CPAP, BiPAP, or intubation

One life-threatening event occurred. The patient had recovered from bronchiolitis and was discharged from the IPD. Seven days later, the child was readmitted for a post-viral cardiac complication, requiring immediate intensive care treatment. There were no mortalities among readmitted patients.

For children not admitted on their initial ED contact, the total number of recontacts within 12 h was 28 (2.4%), of whom, 14 (1.2%) were then admitted. There was no significant difference in recontacts after guideline implementation (Table 2).

### Admission rates

The overall rate of admissions from the emergency department (ED) to the inpatient department (IPD) was 804/1136 (70.8%) before the intervention and 1244/2091 (59.5%) after the intervention (*p*<0.001). For previously healthy children > 90 days of age, the admission rate was 492/753 (65.3%) before the intervention and 673/1263 (53.3%) after the intervention (*p*<0.001). Table 2 shows admission rates (ED to IPD) on the initial hospital contact and on subsequent contacts, for the total group of children as well as for subgroups.

To test if the change in admission rates was temporary, interrupted time series analysis was performed (Online Resource 3). For previously healthy children > 90 days of age, the intervention gave an odds ratio for admission of 0.81 (CI 0.69–0.95). Considering all children, the intervention gave an odds ratio for admission of 0.88 (CI 0.78–0.99). After the intervention, the effect of time on the admission rate was not significant (previously healthy children > 90 days of age, odds ratio 1.00 (CI 0.99–1.01); all children, odds ratio 0.99 (CI 0.992–1.00)) indicating that the reduction in admission rate was consistent over time.

Crude rates of ED contacts and IPD admissions are presented in Table 2.

### Proportion of recontact

**Fig. 2:**
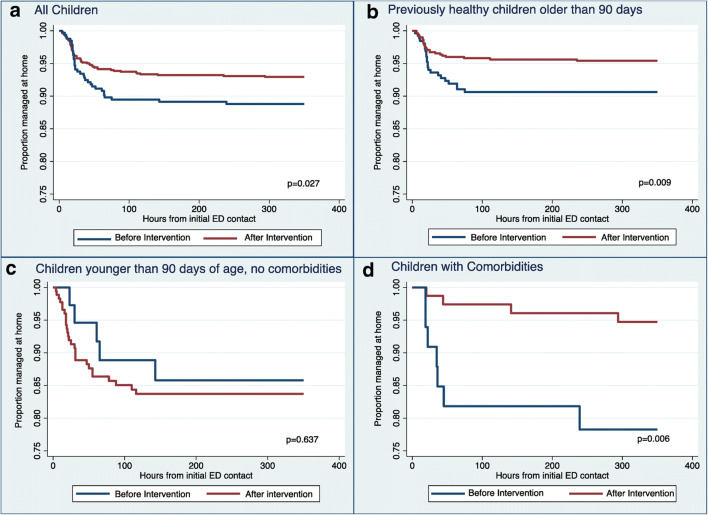
Time to readmission for children not admitted on first ED assessment. Kaplan-Meier plots of children who were not admitted to the ID on their initial contact, comparing the proportion managed at home before vs. after the intervention. Plots are for all children (**a**) and subgroups of children (**b**, **c**, **d**).

A total of 1179 children were discharged from the ED on their initial contact, without admission to the IPD. Proportions of recontact within 14 days (336 h) for VLRTI and leading to hospitalization were plotted as Kaplan-Meier estimates. Children discharged from the ED on their initial contact had significantly less subsequent ED visits leading to hospitalization after the intervention (*p*=0.027). Subgroup analysis showed a similar reduction for previously healthy children > 90 days old (*p*=0.009) and for children with comorbidities (*p*=0.006). In previously healthy children ≤ 90 days old, we found no significant change (Fig. 2).

### Major medical interventions

Children who had their medical records reviewed for major medical interventions had no significant differences in sex, age, or comorbidity before vs. after intervention (Table 1). In admitted, previously healthy children > 90 days, MMI use increased from 22 of 52 (42%) reviewed cases before to 33 of 43 (76%) reviewed cases after guidelines implementation (*p*=0.001) (Table 2).

## Discussion

In this retrospective single-center study, we assessed the value of standardized admission criteria for children under 24 months of age with VLRTI, finding that adverse events did not increase while admission rates decreased. The overall incidence of adverse events in children with VLRTI, who were discharged home after ED assessment, was low both in the total study population and in the primary target group of this study, previously healthy children aged > 90 days. After guidelines implementation, parents were advised how to manage mild respiratory distress, primarily regular removal of nasal secretions by suctioning and adequate intake of fluids. There were no mortalities among patients returning to the hospital. There were no differences in adverse events or readmission within 12 h (defined as unsafe by Luo et al.[27]) after guidelines implementation.

Prior to the analysis, we suspected that our strict guidelines for hospitalization would reduce hospital admissions but increase recontacts and later hospital admissions. Our findings indicate the contrary that the implementation reduced not only IPD admissions on initial contact but also subsequent contacts to the ED. In adult patients, educational sessions are found to effectively improve self-management skills and reduce hospital readmissions [32–34]. The education of parents in nasal suctioning and general management of their sick child, including signs of clinical deterioration, may have increased parental confidence in managing the child at home. It is not possible to differentiate between the specific effect of the guidelines and that of parental education since they were implemented together, but it is likely that more confident parents did play an important role in reducing readmissions.

Guidelines implementation and adherence are often difficult to achieve. We resolved this by an intensive individualized education program and assessment of guidelines adherence in admitted children. Education of the doctors and nurses in the ED and increased focus on VLRTI in general may also have led to more homogeneous care provided on the initial visit, resulting in not only more appropriate hospitalizations but also more appropriate discharges from the initial ED visit. Standardizing patient care can have strong effects on admission parameters. One study found differences in LOS and hospital costs correlated to differences in care practices between hospitals [35]. Another study found reduction in LOS after introduction of discharge criteria [36].

Our guidelines had a positive impact on the overall rate of admission from the ED to the IPD, both on initial visit to the ED and on subsequent visits related to the same illness. As part of the guidelines, a target SpO2 level was introduced. Cunningham et al. found that acceptance of lower targeted SpO2 led to lower admission rates, and patients could be managed on full feeds sooner and had fewer readmissions to hospital [37].

In addition to fewer admissions to the IPD after guidelines introduction, we saw an increased use of major medical interventions among the subset of patients who had their medical records reviewed. Other studies support the use of MMI as a sign of more severe disease, and an increased proportion of patients receiving MMI indicates more appropriate hospitalizations [3, 27, 38]. A reduction in unnecessary hospital admission will not only reduce medical costs but also the potential iatrogenic risk associated with hospitalization of small children with VLRTI [10, 39–41].

The study is limited by its retrospective design, with few clinical variables available, and results may be influenced by unmeasured confounders. In particular, caution should be taken in interpreting results for children with comorbidities predisposing for severe disease, as this is a heterogeneous group with varying degrees of disease severity and often multiple factors that may interact [2, 8, 42, 43]. Studies assessing risk factors for major medical interventions in children with VLRTI often exclude high-risk groups from the analysis [24, 38, 44]. Doctors appointed after the intervention and with experience from other hospitals may make different decisions in the ED [14, 45], a factor that is difficult to account for. However, the reduction in admission rates remained stable over time (ESM 3), suggesting that new doctors either adopted the guidelines or else had a similar practice already. The hospital database did not systematically record parameters such as virology results, frequency and number of treatments given, duration of oxygen therapy, transcutaneous saturation oxygenation (SpO2) in ambient air, or number of daily feeds. A prospective data collection design, including qualitative assessment of junior doctors’ and nurses’ management practices and experiences, might have given more insight. We cannot exclude that some patients visited other parts of the country during their illness. However, this is unlikely to represent a significant number. Given the structure of the Norwegian health-care system and the number of patients assessed, we consider it unlikely that such situations will affect our results.

We conducted a single-center study, comparing outcome before versus after implementation of admission guidelines. Childhood VLRTI has considerable variability from year to year, with inter-seasonal differences in disease severity [46–50]. The guidelines were not intended for patients in whom the doctor suspected asthma, and they did not specifically define VLRTI or asthma. We cannot document why treating physicians diagnosed VLRTI instead of acute asthma, but recommendations at our site state that asthma may be considered from about 12 months of age in children with recurrent wheeze, especially if other atopic diseases or a family history of atopy. Since diagnostic decisions were not the focus of the guidelines or this study, we do not believe that occurrences of misdiagnosis will bias our results. However, differences in assessment and diagnosis of VLRTI between our site and other hospitals may affect replicability [14, 45].

A prospective study design with randomization of different hospitals to either guidelines or standard practice may have better assessed the efficacy of the guidelines, as this would control for differences in disease severity between different seasons and for differences in practice between clinical units.

Data from 2008 was omitted because of significantly higher admission rates compared to the other pre-intervention years, possibly caused by coding issues in the hospital database after moving site. This may introduce bias. However, since admission rates were higher during 2008, including this data would increase the strength of our findings. We therefore consider that this omission does not affect our conclusions.

The total number of adverse events was small, meaning the study did not have enough power to determine whether the guidelines significantly changed the occurrence of adverse events. However, the low number of adverse events indicates that the management of VLRTI among children < 2 years of age was safe, both before and after the introduction of admission guidelines.

In summary, we find that the implementation of our admission guidelines reduced both the admission rate and the return rate of previously healthy children both under and over 90 days of age with VLRTI. There were few adverse events.

## Supplementary information

Online Resource 1 (XLSX 9 kb)

Online Resource 2 (XLSX 11 kb)

Online Resource 3 (PPTX 207 kb)

## Data Availability

Not applicable.

## Abbreviations

BiPAP: Bi-phasic positive airways pressure
CPAP: Continuous positive airway pressure
ED: Emergency department
HFNC: High-flow nasal cannula
IPD: Inpatient department
MMI: Major medical intervention
VLRTI: Viral lower respiratory tract infection
