# Correction to: Viral lower respiratory tract infections—strict admission guidelines for young children can safely reduce admissions

**DOI:** 10.1007/s00431-022-04780-6

**Published:** 2023-01-10

**Authors:** Lise Beier Havdal, Britt Nakstad, Hans Olav Fjærli, Christian Ness, Christopher Inchley

**Affiliations:** 1 Department of Pediatric and Adolescent Medicine, Akershus University Hospital, Sykehusveien 25, 1478 Nordbyhagen, Norway; 2 Division of Paediatric and Adolescent Medicine, Institute of Clinical Medicine, University of Oslo, Oslo, Norway; 3 Institute of Clinical Medicine, University of Oslo, Oslo, Norway

**Correction to: European Journal of Pediatrics (2021) 180:2473–2483** 10.1007/s00431-021-04057-4

In the original published version of the above article, another affiliation has been added to the first author. The affiliations of Lise Beier Havda are as follows:

1 Department of Pediatric and Adolescent Medicine, Akershus University Hospital, Sykehusveien 25, 1478, Nordbyhagen, Norway.

2 Division of Paediatric and Adolescent Medicine, Institute of Clinical Medicine, University of Oslo, Norway.

The original article has been corrected.

