# Supplementary material for: Viral lower respiratory tract infections—strict admission guidelines for young children can safely reduce admissions
Source: Eur J Pediatr. 2021 Apr 8;180(8):2473–83. doi: 10.1007/s00431-021-04057-4 (PMC8285352; doi:10.1007/s00431-021-04057-4)
Supplement: Supplementary file 3 — (PPTX 207 kb) [file 431_2021_4057_MOESM3_ESM.pptx]

## Slide 1
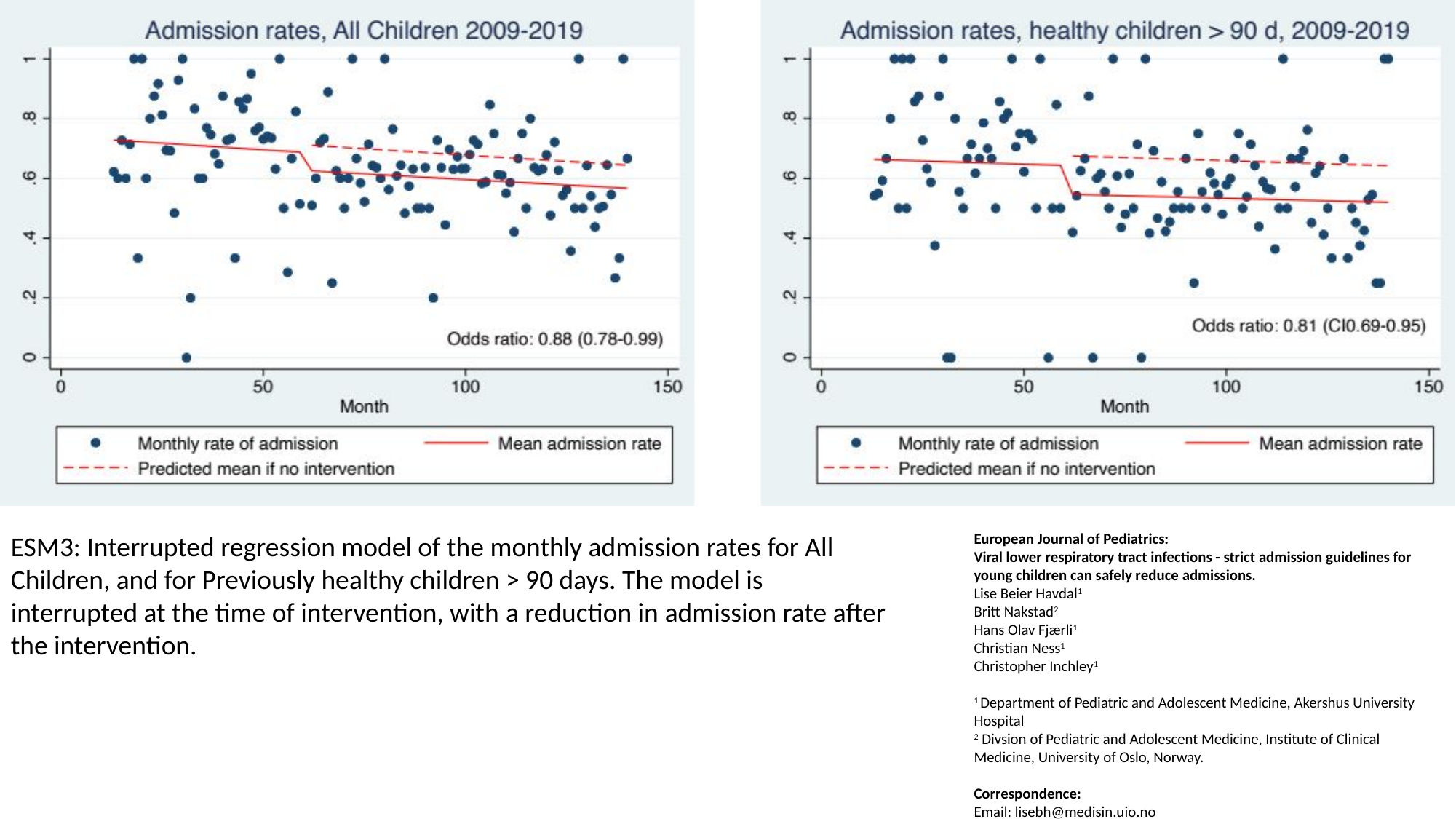

ESM3: Interrupted regression model of the monthly admission rates for All Children, and for Previously healthy children > 90 days. The model is interrupted at the time of intervention, with a reduction in admission rate after the intervention.
European Journal of Pediatrics:
Viral lower respiratory tract infections - strict admission guidelines for young children can safely reduce admissions.
Lise Beier Havdal1
Britt Nakstad2
Hans Olav Fjærli1
Christian Ness1
Christopher Inchley1
1 Department of Pediatric and Adolescent Medicine, Akershus University Hospital
2 Divsion of Pediatric and Adolescent Medicine, Institute of Clinical Medicine, University of Oslo, Norway.
Correspondence:
Email: lisebh@medisin.uio.no
